# Using the Bernoulli trial approaches for detecting ordered alternatives

**DOI:** 10.1186/1471-2288-13-148

**Published:** 2013-12-05

**Authors:** Chia-Hao Chang, Chih-Chien Chin, Weichieh Wayne Yu, Ying-Yu Huang

**Affiliations:** 1 Department of Nursing, Chang Gung University of Science and Technology, Chiayi Campus, Chiayi, Taiwan 61363; 2 Department of Surgery, Section of Colon and Rectal Surgery, Chang Gung Memorial Hospital, Chiayi, Taiwan

## Abstract

**Background:**

Diagnostic problems in clinical trials are sometimes ordinal. For example, colon tumor staging was performed according to the TNM classification. However, clinical data are limited by markedly small sample sizes in some stage.

**Methods:**

We propose a distribution-free test for detecting ordered alternatives in a completely randomized design. The new statistic is based on summing all correctly (ascending) ordered samples.

**Results:**

The exact mean and variance of the null distribution are derived and it is shown that this distribution is asymptotically normal. Furthermore, we show using Monte Carlo simulation that the proposed test is a significant improvement over the Terpstra-Magel test. That is, power is decreased where the investigator falsely assumes an a priori ordering relationship.

**Conclusions:**

We conclude that these tests frequently detect an ordered trend when, in fact, one does not exist. However, the new test can reduce the error rate, at least not to the extent in which the Jonckheere-Terpstra test does.

## Background

This paper focuses on considering nonparametric tests for the non-decreasing ordered alternative of *k*(≥3) groups. The hypothesis to be tested is *H*_0_ : *F*_1_(*x*) = *F*_2_(*x*) = ⋯ = *F*_*k*_(*x*) for all x and *H*_1_ : *F*_1_(*x*) ≥ *F*_2_(*x*) ≥ ⋯ ≥ *F*_*k*_(*x*), for all x with *F*_1_(*x*) > *F*_*k*_(*x*) for some x, where *F*_1_(*x*), *F*_2_(*x*), ⋯, *F*_*k*_(*x*) are continuous distribution functions.

In this article, we assume the location model with *F*_*i*_(*x*) = *F*(*x* - *μ* - *θ*_*i*_), where *μ* is a location parameter and *θ*_*i*_ represents the effect of group *i*, *i* = 1, 2, …, k. This implies that the underlying populations may differ only in location. Throughout the article, let $x_{i1},\;x_{i2},\ldots ,x_{in_{i}},\;i\;=\;1,\;2,\;\ldots ,$ k represent independent random samples from the *k* populations with distribution functions *F*_*i*_ (*x*), *i* = 1, 2, …, k, respectively.

Nonparametric order restricted inference has been extensively investigated in past literature and new studies are continuing to emerge. For instance, Puri [1], Puri and Sen [2], and Padmanabhan et al. [3] applied the concept of Chernoff–Savage-type statistics to nonparametric ordered alternative tests. Studies that used power results to compare the validity of linear rank tests included Büning and Kössler [4], Beier and Büning [5], Büning and Kössler [6], Büning [7], Büning and Kössler [8], Büning and Kössler [9], Kössler [10] and Kössler [11].

The earliest and most classic treatment of *k* (≥ 3)-sample distribution-free statistic for ordered alternatives was proposed by Jonckheere [12] and Terpstra [13]. The test is known as the Jonckheere-Terpstra test (hereafter referred to as the JT test) and is based on a sum of $C_{2}^{k}$ Mann–Whitney statistics (Mann and Whitney, [14]; Hollander and Wolfe, [15]). In order to define the JT statistic, we express the Mann–Whitney statistics as

$$U_{\mathrm{lm}}=\sum_{j_{l}=1}^{n_{l}}\sum_{j_{m}=1}^{n_{m}}I\left(x_{lj_{l}},x_{mj_{m}}\right),\;1\leq l<m\leq k,$$

where $I\left(x_{lj_{l}},x_{mj_{m}}\right)=\left\{{}_{0,\;\text{otherwise}\;}^{1,\;\begin{array}{cc} \mathrm{if} & x_{lj_{l}}<x_{mj_{m}} \end{array}}\right)$, and the JT statistic is given by $\mathrm{JT}=\sum_{l=1}^{k-1}\sum_{m=l+1}^{k}U_{\mathrm{lm}}$.

Other tests for ordered alternatives were developed by Cuzick [16] and Le [17]. Among the JT, Cuzick, and Le tests, the results from Mahrer and Magel [18] did not establish any of the tests as having overwhelmingly higher power over the others across different location parameters. Neuhauser et al. [19] presented a modified version of the JT test (hereafter referred to as the MJT test). The form of the MJT statistic is identical to the JT test except that the Mann–Whitney statistic *U*_*lm*_ multiplies the weight *m - l* as the new kernel. Study results showed that the MJT test often produced a higher power than the JT test for the ordered alternative. We also noted that Tryon and Hettmansperger [20] presented the JT and MJT tests as members of a more general class of nonparametric tests. In the test statistics described above the kernels of the tests are almost all derived by comparing two pairs of sample observations at a time. However, Terpstra and Magel [21] proposed a test (hereafter referred to as the TM test) where the kernels of TM test are based on information obtained simultaneously across all samples. The statistic is determined by adding the $\prod_{i=1}^{k}n_{i}$ indicator functions, that is,

$$\mathrm{TM}=\sum_{j_{1}=1}^{n_{1}}\cdots \sum_{j_{k}=1}^{n_{k}}I\left(x_{1j_{1}}\leq x_{2j_{2}}\leq \cdots \leq x_{kj_{k}}\right)$$

where $I\left(x_{1j_{1}}\leq x_{2j_{2}}\leq \cdots \leq x_{kj_{k}}\right)$ is equal to one, provided at least one strict inequality; otherwise, $I\left(x_{1j_{1}}\leq x_{2j_{2}}\leq \cdots \leq x_{kj_{k}}\right)$ is equal to zero..

Terpstra et al. ([22]a, b) proposed a new nonparametric test statistic (hereafter referred to as the KTP test) that is a generalization of the TM test. The idea is to replace the indicator kernel from the TM test with Spearman’s rank correlation coefficient, that is,

$$\mathrm{KTP}=\sum_{j_{1}=1}^{n_{1}}\cdots \sum_{j_{k}=1}^{n_{k}}r\left(x_{1j_{1}},x_{2j_{2}},\ldots ,x_{kj_{k}}\right),$$

where is Spearman’s rank correlation coefficient between the observed data and the corresponding group number.

In this study, we propose a new test is based on the information present in the $N^{*}=\prod_{i=1}^{k}n_{i}$ k-tuplets, where a k-tuplet includes one observation from each treatment group. All correctly (ascending) ordered samples are then summed to form a statistic that is distributed approximately as a normal distribution. Details of this new test and its asymptotic distribution are provided, and the computational algorithm is presented in the Additional file 1. A colon cancer data example is given in data example section. Finally, we present a finite sample simulation study which compares the proposed test, the JT test, MJT test, TM test, and the KTP test in terms of power. A computer program written in R that implements the proposed methods will be available from the first author upon request. It is recommended that readers who are not interested in the details of the computational algorithm skip the Additional file 1.

## Methods

### Test statistic

The new nonparametric test for non-decreasing alternatives is based on the following statistic,

$$T=\sum_{j_{1}=1}^{n_{1}}\cdots \sum_{j_{k}=1}^{n_{k}}k\left(x_{1j_{1}},x_{2j_{2}},\ldots ,x_{kj_{k}}\right),$$

Where $k\left(x_{1},x_{2},\ldots ,x_{k}\right)=\sum_{i=1}^{k}I\left(R\left(x_{i}\right)=i\right)$, R (x_i_) denotes the rank of x_i_ with respect to x_1_, x_2_,…, x_k_, and I(.) denotes the indicator function.

The remainder of this section presents and derives results pertaining to the null distribution of the proposed test statistic. We assume throughout this section that the observed data, {X_ij_} is essentially a random sample from some continuous probability distribution function F. Hence, the possibility of ties has a probability of zero. In principle the test statistic uses the k-tuplet method of Terpstra and Magel. Additionally, in the null hypothesis each $k\left(x_{1j_{1}},x_{2j_{2}},\ldots ,x_{kj_{k}}\right)$ follows the Binomial (k, 1/k) distribution. For these reasons, we will refer to this test as the KTMB test.

### The exact null distribution

Let N denote the sum of the sample sizes for each treatment. Namely, let *N* = *n*_1_ + ⋯ + *n*_*k*_. Here, we have *N* !/(*n*_1_ ! ⋯ *n*_*k*_ !) partitions of the numbers 1, …, N. The null distribution of T means each one of these partitions is equally likely so the mean and variance can be calculated directly by multiplying each possible value of T with its probability. When the number of partitions is small, we can easily calculate the exact distribution by hand or with the computer. Table 1 shows the probabilities, means, and variances of the test statistic T for sample size arrangements (2, 1, 1), (2, 1, 2), and (1, 1, 3) respectively.

**Table 1:** Some exact null distributions for the proposed test statistic

**Sample sizes**	**Test statistic value (KTMB)**											**Mean and variance**	
(n_1_, n_2_, n_3_)	**0**	**1**	**2**	**3**	**4**	**5**	**6**	**7**	**8**	**9**	**12**	E [T]	V[T]
(2, 1, 1)	2/12	3/12	4/12	1/12	1/12	-	1/12	-	-	-	-	2	2.667
(2, 1, 2)	2/30	2/30	6/30	4/30	6/30	2/30	3/30	2/30	2/30	-	1/30	4	6.867
(1, 1, 3)	2/20	3/20	4/20	6/20	1/20	1/20	1/20	1/20	-	1/20	-	3	5.000

Real world cases are not always as simple as the above illustration. For example, when k=4, *n*_1_ = *n*_2_ = *n*_3_ = *n*_4_ = 5, we have 20 !/(5 ! 5 ! 5 ! 5 !) = 1.1733 × 10^10^ partitions. Such a distribution function cannot be calculated even with the most efficient personal computers. We will therefore introduce a Monte Carlo approximation to the null distribution. On the other hand, if the distribution of T can be approximated or can be shown to converge to a well-known distribution, we can avoid computational complexity altogether.

### The mean and variance

If the asymptotic null distribution of a test statistic is normal and the exact mean and variance of T under H_0_ in standard form can be established we can then standardize T by using the exact mean and variance to obtain *Z*_*KTMB*_, where ZKTMB=T-E0T/VT0. In this case, we can find critical values from the standard normal table.

We will start by finding the mean value of T, E_0_(T), noting that T is nothing but a sum of the $k\times \prod_{i=1}^{k}n_{i}$ Bernoulli (1/*k*) distribution. It is straightforward to get

(1) $$E_{0}\left(T\right)=\prod_{i=1}^{k}n_{i}$$

Here, and in the following, we let $n^{*}=\prod_{i=1}^{k}n_{i},$

(2) $$V_{0}\left(T\right)=v_{0}^{2}+\sum_{i=1}^{k-1}v_{i}^{2}+v_{k}^{2}$$

where $v_{0}^{2}=\left\{1-\left(1/k\right)\right\}\prod_{i=1}^{k}n_{i}$ for no tie, $v_{k}^{2}=\;n^{*}k\left(k-1\right)\left\{\left(k-2\right)!/k!-1/k^{2}\right\}$ for k ties for i ≠ j.

For the case of *i* ties, we present an algorithm for the computation of the $\sum_{i=1}^{k-1}v_{i}^{2}$ in Additional file 1. Readers who are not interested in the details of this algorithm may want to skip the Additional file 1 and go to data example section, in which examples based on real data are provided.

### The asymptotic null distribution

In this section we will look to see if the asymptotic null distribution of test statistic *T* follows the standard normal distribution. In other words, we prove that

(3) $$T^{*}=\frac{T-E_{0}\left(T\right)}{\sqrt{V_{0}\left(T\right)}}\overset{D}{\to }N\left(0,1\right).$$

H_0_ will therefore be rejected for large values of T^*^. The normal approximation for the procedure is to reject *H*_*0*_ if T* ≥ *z*_1 - *α*_; otherwise do not reject *H*_*0*_. Note that the critical value *z*_1 - *α*_ is chosen to make the Type I error probability equal to *α*. That is, *α* ≈ *P*(T* ≥ *z*_1 - *α*_|*H*_0_ true). We note that (3) is a direct consequence of Theorem 1, which we now state.

Theorem 1 *Let*$N=\sum_{l=1}^{k}n_{l}$*and assume*$\frac{n_{l}}{N}=\lambda_{l}+o\left(1\right)$*where λ*_*l*_ ∈ (0, 1) *. Then, under*$H_{0,\;}T_{N}\overset{\mathrm{def}}{=}\frac{1}{k\cdot N^{k-1/2}}\sum_{j_{1}=1}^{n_{1}}\cdots \sum_{j_{k}=1}^{n_{k}}\left[k\left(x_{1j_{1}},\cdots ,x_{kj_{k}}\right)-1\right]\overset{D}{\to }N\left(0,\sum_{l=1}^{k}\lambda_{l}^{*}\sigma_{\mathrm{lk}}^{2}\right),$*where*$\lambda_{l}^{*}=\lambda_{l}\prod_{j=1}^{k}\lambda_{j}^{2I\left(j\neq l\right)}.$

*Proof of Theorem 1* Terpstra and Magel [21] proved that *TM* statistic follows a normal distribution as sample sizes go to infinity by using projection technique from Hettmansperger and McKean ([23], p. 81). In what follows all limits are taken with respect to *N*, as *N→∞*. To apply their theorem to our case, let

$$\begin{array}{ll} E\left[T_{N}|X_{\mathrm{lm}}\right] & =\frac{1}{k\cdot N^{k-1/2}}\sum_{j_{1}=1}^{n_{1}}\cdots \sum_{j_{k}=1}^{n_{k}}\sum_{i=1}^{k}[I_{i}(j_{l}=m)Z_{\mathrm{lk}}\\ & \times (X_{\mathrm{lm}})+I_{i}(j_{l}\neq m)\frac{1}{k}-\frac{1}{k}]=\frac{L_{n}\left(l\right)}{N^{k-1/2}}[Z_{\mathrm{lk}}\left(X_{\mathrm{lm}}\right)-\frac{1}{k}] \end{array}$$

$$Z_{\mathrm{lk}}\left(X_{\mathrm{lm}}\right)\overset{\mathrm{def}}{=}\frac{\left(k-1\right)!}{\left(l-1\right)!\left(k-l\right)!}F^{l-1}(x){\left[1-F\left(x\right)\right]}^{k-l},\;l\;=\;1,\ldots ,\;k.$$

where

$$L_{n}\left(l\right)=\prod_{j=1}^{k}n_{j}^{I\left(j\neq l\right)}.$$

The projection of *T*_*N*_, say, *P*_*N*_ can be defined as,

(4) $$\begin{array}{ll} P_{N} & =\sum_{l=1}^{k}\sum_{m=1}^{n_{l}}E\left[T_{N}|X_{\mathrm{lm}}\right]\\ & =\sum_{l=1}^{k}\left[\frac{L_{n}\left(l\right)\sqrt{n_{l}}}{N^{k-1/2}}\right]\frac{1}{\sqrt{n_{l}}}\sum_{m=1}^{n_{l}}\left[Z_{\mathrm{lk}}\left(X_{\mathrm{lm}}\right)-\frac{1}{k}\right]. \end{array}$$

$E\left[Z_{\mathrm{lk}}\left(X_{\mathrm{lm}}\right)\right]=\frac{1}{k}$ and $V\left[Z_{\mathrm{lk}}\left(X_{\mathrm{lm}}\right)\right]=\sigma_{\mathrm{lk}}^{2}$ can be proved by Beta distribution. The convergence criteria on the *k* sample sizes imply that,

(5) $$\frac{L_{n}\left(l\right)\sqrt{n_{l}}}{N^{k-1/2}}=\sqrt{\lambda_{l}^{*}}+o\left(1\right).$$

It now follows from (4), (5), and limiting moment generating function theory that,

(6) $$P_{N}\overset{D}{\to }N\left(0,\sum_{l=1}^{k}\lambda_{l}^{*}\sigma_{\mathrm{lk}}^{2}\right).$$

Let us now consider *V* [*T*_*N*_], which we write as,

(7) $$\begin{array}{l} V\left[T_{N}\right]=\frac{1}{k^{2}N^{2k-1}}\sum_{i_{1}=1}^{n_{1}}\cdots \sum_{i_{k}=1}^{n_{k}}\sum_{j_{1}=1}^{n_{1}}\cdots \sum_{j_{k}=1}^{n_{k}}\\ \times \mathrm{COV}\left[k\left(x_{1i_{1}},\cdots ,x_{ki_{k}}\right),k\left(x_{1j_{1}},\cdots ,x_{kj_{k}}\right)\right]. \end{array}$$

Consider first the case of *k* ties for *i* ≠ *j*. it is straightforward to show $\mathrm{COV}\left[k\left(x_{1i_{1}},\cdots ,x_{ki_{k}}\right),\;k\left(x_{1j_{1}},\cdots ,x_{kj_{k}}\right)\right]=k\left(k-1\right)\left[\frac{\left(k-2\right)!}{k!}-\frac{1}{k^{2}}\right]$. Next, consider the case in which there are exactly three ties among the different subscripts. For example, if we let *R*_*u*_ denotes the rank of *x*_*u*_ with respect to *x*_*1*_, *x*_*2*_,…, *x*_*k*_, *R*_*v*_ denotes the rank of *x*_*v*_ with respect to *x*_*1*_, *x*_*2*_,…, *x*_*2k-3*_, *u* < *v*-*k*, *R*_*u*_*< R*_*v*_, and *X*_*1*_, *X*_*2*_, *X*_*3*_ denote the tied observations then the covariance term has the form *COV [I*_*u*_, *I*_*v*_*]* where, *R*_*u*_ denotes the rank of *x*_*u*_ with respect to $X_{4},\ldots ,X_{l_{1}},\;X_{1},X_{l_{1}+1},\ldots ,X_{u},\ldots ,X_{l_{2}},\;X_{2},X_{l_{2}+1},\ldots ,X_{l_{3}},\;X_{3},X_{l_{3}+1}\ldots ,X_{k}$ and *I*_*u*_ = *I*(*R*_*u*_ = *u*), and *R*_*v*_ denotes the rank of *x*_*v*_ with respect to $X_{k+1},\ldots ,X_{k+l_{1}-3},X_{1},X_{k+l_{1}-2},\ldots ,X_{k+l_{2}-3},X_{2},X_{k+l_{2}-2},\ldots ,X_{v},\ldots ,X_{k+l_{3}-3},X_{3},X_{k+l_{3}-2},\ldots ,X_{2k-3}$ and *I*_*v*_ = *I*(*R*_*v*_ = *v*).

Under *H*_*0*_, $E\left[I_{u}\right]=E\left[I_{v}\right]=\frac{1}{k}$*.* Next, consider *E [I*_*u*_*I*_*v*_*]*. This expectation contains 2*k*-3 observations, so that under *H*_*0*_, each of the (2*k*-3)! permutations of the observations are equally likely. However, there are only the numbers of *{1 : R*_*u*_*-1}∩{1 : R*_*v*_*-1}* possible ways, say *I*_*SS*_(*R*_*u*_, *R*_*v*_), plus the numbers of *{R*_*u*_*+1 : 2k-3}∩{1 : R*_*v*_*-1}* possible ways, say *I*_*LS*_(*R*_*u*_, *R*_*v*_), plus the numbers of *{R*_*u*_*+1 : 2k-3}∩{R*_*v*_*+1 : 2k-3}* possible ways, say *I*_*LL*_(*R*_*u*_, *R*_*v*_), to preserve *X*_*1*_, *X*_*2*_, and *X*_*3*_. Furthermore, there are $C_{u-4}^{I_{\mathrm{SS}}\left(R_{u},R_{v}\right)-t_{1}}$ possible ways to preserve $X_{4},\ldots ,X_{l_{1}},X_{l_{1}+1},\ldots ,X_{u-1}$ to the left of *X*_*u*_, $C_{v-k-1-\left(u-4\right)}^{I_{\mathrm{LS}}\left(R_{u},R_{v}\right)-t_{2}}$ possible ways to preserve $X_{k+1},\ldots ,X_{k+l_{1}-3},X_{k+l_{1}-2},\ldots ,X_{k+l_{2}-3},X_{k+l_{2}-2},\;X_{v-1}$ to the left of *X*_*v*_, $C_{k-u-\left\{I_{\mathrm{LS}}-1-\left[v-k-1-\left(u-4\right)\right]\right\}}^{I_{\mathrm{LL}}\left(R_{u},R_{v}\right)-t_{3}}$ possible ways to preserve $X_{u+1},\ldots ,X_{l_{2}},X_{l_{2}+1},\ldots ,X_{l_{3}},\;X_{l_{3}+1}\ldots ,X_{k}$ to the right of *X*_*u*_, and $C_{I_{\mathrm{LL}}\left(R_{u},R_{v}\right)-1-\left(k-u-I_{\mathrm{LS}}+1+v-k-1-u+4\right)}^{I_{\mathrm{LL}}\left(R_{u},R_{v}\right)-1-\left(k-u-I_{\mathrm{LS}}+1+v-k-1-u+4\right)}=1$ possible way to preserve $X_{v+1},\ldots ,X_{k+l_{3}-3},X_{k+l_{3}-2},\ldots X_{2k-3}$ to the right of *X*_*v*_. Hence, these arguments imply that,

$$\mathrm{COV}\left(I_{u},I_{v}\right)=\frac{3!\left(k-4\right)!\left(k-4\right)!C_{t_{1}+t_{2}+t_{3}}^{I_{\mathrm{SS}}\left(R_{u},R_{v}\right)+I_{\mathrm{LS}}\left(R_{u},R_{v}\right)+I_{\mathrm{LL}}\left(R_{u},R_{v}\right)}\cdot C_{u-4}^{I_{\mathrm{SS}}\left(R_{u},R_{v}\right)-t_{1}}}{\left(2k-3\right)!}\frac{C_{v-k-1-\left(u-4\right)}^{I_{\mathrm{LS}}\left(R_{u},R_{v}\right)-t_{2}}C_{k-u-\left\{I_{\mathrm{LS}}\left(R_{u},R_{v}\right)-1-\left[v-k-1-\left(u-4\right)\right]\right\}}^{I_{\mathrm{LL}}\left(R_{u},R_{v}\right)-t_{3}}}{}-\frac{1}{k^{2}}.$$

where *t*_*1*_ + *t*_*2*_ + *t*_*3*_ = 3, *t*_*1*_, *t*_*2*_, and *t*_*3*_ = 0, 1, 2, 3.

Now, for a given *l*_*1*_, *l*_*2*_, and *l*_*3*_, there are $n_{l_{1}}n_{l_{2}}n_{l_{3}}\prod_{t=1}^{k}{\left[n_{t}\left(n_{t}-1\right)\right]}^{I\left(t\neq l_{1}\right)I\left(t\neq l_{2}\right)I\left(t\neq l_{3}\right)}$ of these covariance terms. Next, consider all possible *R*_*u*_, *R*_*v*_ and all possible treatment locations (*m* and *n*), in the case of one tie *X*_*1*_, (7) reduces to,

(8) $$\begin{array}{l} \sum_{l=1}^{k}\frac{n_{l}\prod_{t=1}^{k}{\left[n_{t}\left(n_{t}-1\right)\right]}^{I\left(t\neq l\right)}}{N^{2k-1}}\sum_{R_{u}=m}^{k-i+m}\sum_{R_{v}=n}^{k-i+n}[\frac{C_{t_{1}+t_{2}+t_{3}}^{I_{\mathrm{SS}}\left(R_{u},R_{v}\right)+I_{\mathrm{LS}}\left(R_{u},R_{v}\right)+I_{\mathrm{LL}}\left(R_{u},R_{v}\right)}}{1}\\ \times \frac{\left(k-2\right)!\left(k-2\right)!C_{u-4}^{I_{\mathrm{SS}}\left(R_{u},R_{v}\right)-t_{1}}C_{v-k-1-\left(u-4\right)}^{I_{\mathrm{LS}}\left(R_{u},R_{v}\right)-t_{2}}C_{k-u-\left\{I_{\mathrm{LS}}\left(R_{u},R_{v}\right)-t_{2}-\left[v-k-1-\left(u-4\right)\right]\right\}}^{I_{\mathrm{LL}}\left(R_{u},R_{v}\right)-t_{3}}}{\left(2k-1\right)!}-\frac{1}{k^{2}}]\\ =\sum_{l=1}^{k}\lambda_{l}^{*}\sigma_{\mathrm{lk}}^{2}+o\left(1\right) \end{array}$$

where *t*_*1*_ + *t*_*2*_ + *t*_*3*_ = 1, *t*_*1*_, *t*_*2*_, and *t*_*3*_ = 0, 1.

From (6) and (8) it follows that *V*[*T*_*N*_] - *V*[*P*_*N*_] = *o*(1). Asymptotic normality results are attainable.

### Patient characteristics

The institutional review board of Chang Gung Memorial Hospital approved the present study. Detailed information about patients with colon cancer, such as patient- and tumor-related factors and follow-up status, was retrieved from the Colorectal Section Tumor Registry at Chang Gung Memorial Hospital, Taiwan. All the data in this registry were prospectively collected.

## Results and discussion

### Data examples

Between January 2006 and December 2010, 154 consecutive patients with histologically confirmed colonic adenocarcinoma underwent curative surgeries at the Chang Gung Memorial Hospital in Chiayi. The stage IV colon cancer, non-curative surgeries, rectal cancer and mucinous adenocarcinomawere excluded in this study. Tumor staging was performed according to the TNM classification described in the 6th edition of the cancer staging manual of the American Joint Committee on Cancer (Stage I, II, IIIA and IIIB). The different tumor staging require a different treatment to optimize patient and hospital outcomes. An ordinal logistic regression model was developed with predictors as follows: age, gender, tumor location, histologic differentiation, preoperative albumin level, preoperative carcinoembryonic antigen level, and underlying medical illnesses.

To illustrate the KTMB test, assume an outcome with four stages and a set of cases consisting of one case from each stage. The case from Stage I has risks of 0.50, 0.25, 0.15 and 0.10 for Stage I, II, IIIA and IIIB, respectively. The case from Stage II has risks 0.26, 0.52, 0.17 and 0.05; the case from Stage IIIA has risks 0.06, 0.32, 0.42 and 0.20; the case from Stage IIIB has risks 0.12, 0.18, 0.30 and 0.40. The risk for Stage IIIB (say, event) is higher for the case that belongs to this stage (0.40) than for the other cases (0.10, 0.05 and 0.20). The risk for event is second-highest for the case from Stage IIIA (0.20 versus 0.10, 0.05 and 0.40). However, the risk for event is lowest for the case from Stage II (0.05 versus 0.10, 0.20 and 0.40). The risk for event is third-highest for the case from Stage I (0.10 versus 0.05, 0.20 and 0.40). Therefore, the risks correctly identify the cases from Stage IIIA and IIIB but not Stage I and II, resulting in a score of 2 for this set (*k*(*x*_1_, *x*_2_, *x*_3_, *x*_4_)).

Hence, the set of hypotheses was *H*_0_ : *F*_*I*_(*x*) = *F*_*II*_(*x*) = *F*_*IIIA*_(*x*) = *F*_*IIIB*_(*x*) for all x and *H*_1_ : *F*_*I*_(*x*) ≥ *F*_*II*_(*x*) ≥ *F*_*IIIA*_(*x*) ≥ *F*_*IIIB*_(*x*), where *F*_*I*_(*x*) ≠ *F*_*IIIB*_(*x*) for some x.

**Figure 1:**
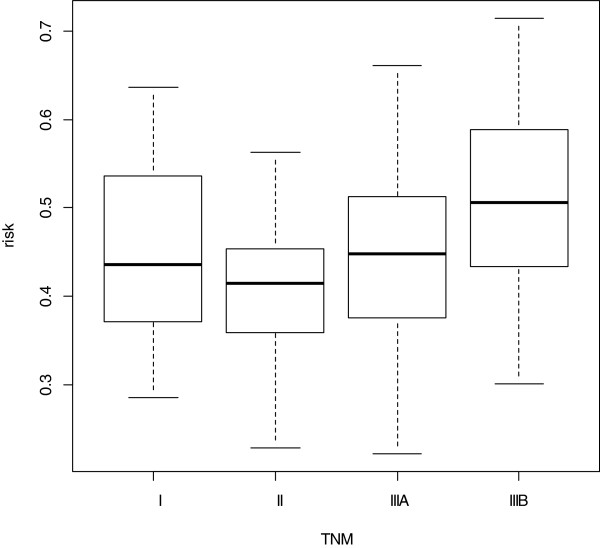
Box plots of risk for Stage IIIB versus TNM for clinical data.

Five test statistics and the corresponding p-values are given in Table 2. Since a plot of this data set in Figure 1 exhibits a non-increased trend, it appears that the JT, MJT and KTP tests have falsely conclusion (p < 0.05). The KTMB test has the largest p-value (See Table 2). Moreover, Stage IIIB patients reported significantly more risk for Stage IIIB than Stage II subjects (ANOVA, post hoc: IIIB > II, p = 0.003) while Stage I, II and IIIA patients did not differ in risk for Stage IIIB (ANOVA, post-hoc: p>0.05) Hence, we conclude that the risk for Stage IIIB do not increase with the patient’s TNM in the model. That is, the discrimination performance of the ordinal logistic model is not very well between Stage I, II and IIIA.

**Table 2:** Order restricted inference results for the colon cancer data

	**JT**	**MJT**	**TM**	**KTP**	**KTMB**
Test Statistic	3.01	2.69	1.44	2.17	1.29
p-value	0.00132	0.00361	0.07473	0.01483	0.09830

### Comparison with respect to size and power

To determine if the underlying population came from different skew and kurtosis distributions that impact on the power of the test statistic, we used log-F distributions with combinations of 2, 4.5 and 10 degrees of freedom to generate the random variable. We can therefore define random variable *X*_*ij*_ as: *X*_*ij*_ = *θ*_*i*_ + *ϵ*_*ij*_, where *ϵ*_*ij*_ is the iid log-F distribution, and *θ*_*i*_ are location parameters.

For the numbers of treatment (k), sample sizes (*n*_*i*_) and location parameters (*θ*_*i*_) we examine the different combinations of k = 3 and 4, n_i_ = 4, 5, 8 and 10, *θ*_*i*_ = 0, 0.25, 0.5, 0.75, 1 and 1.25. We investigated designs under assumed alternatives which are of the forms of concave and convex. Programs to compare powers were written in R 2.9.2 (R Development Core Team, Vienna, Austria). The estimations were conducted by simulating 10,000 different sets of samples. Furthermore, we estimated the power by counting the number of times H_0_ was rejected and using the value to divide by 10,000. Ideally, we believe that the test should have higher power than a general alternative test when H_1_ is true, and should have low power for any alternative that does not fit the profile given in H_1_.

In general, the JT and KTP tests have the highest powers for the ordered alternative cases. Comparing with TM test, the gain percentage in power, DP = (KTMB - TM)/TM, ranges from -6.29% to 11.27% with the average gain percentage in power being 2.63% (difference of percentage).

Consider the corresponding alternatives of the form of concave and convex shapes. The powers of the KTMB test outperforms (lower power) the KTP, JT, MJT, and TM tests when balanced design. The loss percentage in power, DP = (minimum of KTP, JT, MJT, and TM – KTMB)/KTMB, ranges from -7.28% to 20.00% with the average loss percentage in power being 4.25% for *k* = 3. The DP ranges from -13.2% to 27.68% with the average loss percentage in power being 8.83% for k = 4.

When the sample sizes corresponding to the non-decreased trend location parameters are comparatively large, the KTMB test is better than KTP, MJT, JT, and TM tests. The DP ranges from -7.29% to 9.30% with the average loss percentage in power being 2.47% for k = 3. The DP ranges from -6.52% to 30.93% with the average loss percentage in power being 12.07% for k = 4. However, the KTP test slightly better than KTMB test when the underlying population is skewed to the right (see Table 3).

**Table 3:** Estimated powers and type I error rates of ordered tests under significance level 0.05

**Location parameter**	***KTMB***	***KTP***	***JT***	***MJT***	***TM***	***DP***	**Location parameter**	***KTMB***	***KTP***	***JT***	***MJT***	***TM***	***DP***
**n**_**1**_**=4, n**_**2**_**=4, n**_**3**_**=4**							**n**_**1**_**=10, n**_**2**_**=10, n**_**3**_**=5**						
**Log-F (2,4.5)**							**Log-F (2,4.5)**						
(0, 0, 0)	0.0492	0.0496	0.0489	0.0496	0.0494		(0, 0, 0)	0.0494	0.0481	0.0488	0.0494	0.0509	
(0, 0.25, 0.5)	0.1176	0.1243	0.1096	0.1243	0.1255	-6.29%	(0, 0.25, 0.5)	0.1491	0.1542	0.1566	0.1554	0.1491	0%
(0.5, 0, 0.25)	0.0295	0.0344	0.0295	0.0344	0.0346	0.00%	(0.25, 0.5, 0)	0.0209	0.022	0.0346	0.0316	0.0217	3.83%
(0.25, 0.75, 0)	0.021	0.029	0.0212	0.029	0.0236	0.95%	(0.5, 0.75, 0)	0.0086	0.0094	0.0167	0.0143	0.0096	9.30%
Log-F (4.5, 4.5)							Log- F (4.5, 4.5						
(0, 0, 0)	0.0511	0.0521	0.0518	0.0521	0.0531		(0, 0, 0)	0.0492	0.0478	0.0501	0.0488	0.0512	
(0, 0.25, 0.5)	0.1533	0.1612	0.1447	0.1612	0.1604	-4.43%	(0, 0.25, 0.5)	0.2079	0.2118	0.2181	0.217	0.2021	2.87%
(0.5, 0, 0.25)	0.0189	0.0259	0.0212	0.0259	0.0237	12.17%	(0.25, 0.5, 0)	0.0207	0.0217	0.034	0.0303	0.0215	3.86%
(0.25, 0.75, 0)	0.0207	0.0246	0.0213	0.0246	0.0239	2.90%	(0.5, 0.75, 0)	0.0058	0.0058	0.012	0.0093	0.006	0.00%
Log-F (10, 4.5)							Log-F (10, 4.5)						
(0, 0, 0)	0.0509	0.0523	0.0519	0.0523	0.0527		(0, 0, 0)	0.0488	0.0494	0.0517	0.0505	0.051	
(0, 0.25, 0.5)	0.1802	0.1961	0.1742	0.1961	0.1843	-2.22%	(0, 0.25, 0.5)	0.2522	0.2634	0.27	0.268	0.2446	3.11%
(0.5, 0, 0.25)	0.016	0.021	0.0166	0.021	0.0192	3.75%	(0.25, 0.5, 0)	0.0217	0.0195	0.0353	0.0302	0.024	-10.14%
(0.25, 0.75, 0)	0.0161	0.0196	0.0161	0.0196	0.0198	0.00%	(0.5, 0.75, 0)	0.0064	0.0058	0.0119	0.0093	0.0076	-9.38%
n_1_=4, n_2_=4,							n_1_=8, n_2_=8,						
n_3_=4, n_4_=4							n_3_=8, n_4_=4						
Log-F (2,4.5)							Log-F (2,4.5)						
(0, 0, 0, 0)	0.0512	0.0519	0.0515	0.0519	0.0514		(0, 0, 0, 0)	0.0505	0.0484	0.0518	0.0507	0.0479	
(0, 0.25, 0.5, 0.75)	0.165	0.1806	0.188	0.1806	0.1615	2.17%	(0, 0.25, 0.5, 0.75)	0.2042	0.2313	0.2408	0.2372	0.1915	6.67%
(0.75, 0, 0.25, 0.5)	0.0388	0.0388	0.0417	0.0388	0.0471	0.00%	(0.5, 0.5, 0.5, 0)	0.0121	0.0152	0.0197	0.018	0.013	7.44%
(0.25, 0.75, 1.25, 0)	0.0225	0.0315	0.0313	0.0315	0.0276	22.67%	(0.25, 0.5, 0.75, 0)	0.0258	0.0332	0.0641	0.0575	0.0283	9.69%
Log-F (4.5, 4.5)							Log-F (4.5,4.5)						
(0, 0, 0, 0)	0.0507	0.0503	0.0509	0.0503	0.0508		(0, 0, 0, 0)	0.0505	0.0487	0.0508	0.0477	0.048	
(0, 0.25, 0.5, 0.75)	0.2242	0.2513	0.2606	0.2513	0.2143	4.62%	(0, 0.25, 0.5, 0.75)	0.2987	0.3358	0.3572	0.3562	0.2795	6.87%
(0.75, 0, 0.25, 0.5)	0.0297	0.0282	0.0304	0.0282	0.0385	-5.05%	(0.5, 0.5, 0.5, 0)	0.0092	0.0086	0.0122	0.0111	0.0111	-6.52%
(0.25, 0.75, 1.25, 0)	0.0237	0.0291	0.0298	0.0291	0.0277	16.88%	(0.25, 0.5, 0.75, 0)	0.025	0.0285	0.0714	0.0592	0.0302	14.00%
Log-F (10, 4.5)							Log-F (10, 4.5)						
(0, 0, 0, 0)	0.0527	0.0513	0.0509	0.0513	0.0527		(0, 0, 0, 0)	0.0504	0.0492	0.0493	0.0514	0.0508	
(0, 0.25, 0.5, 0.75)	0.2882	0.3179	0.3316	0.3179	0.259	11.27%	(0, 0.25, 0.5, 0.75)	0.3697	0.4268	0.4489	0.4492	0.3458	6.91%
(0.75, 0, 0.25, 0.5)	0.0168	0.0211	0.0204	0.0211	0.0216	21.43%	(0.5, 0.5, 0.5, 0)	0.0049	0.0055	0.01	0.0079	0.0079	12.24%
(0.25, 0.75, 1.25, 0)	0.025	0.0259	0.029	0.0259	0.0317	3.60%	(0.25, 0.5, 0.75, 0)	0.0273	0.027	0.076	0.0568	0.0328	-1.10%

Based on the simulation results above, we conclude that the KTMB test is better than the TM test in regards to the power against ordered alternatives. Moreover, the KTMB test offers built in protection for the situation when an investigator falsely assumes an a priori ordered relationship.

Table 3 just represent a small subset of the many different scenarios that we simulated. For example, we also conducted simulations for numerous other alternative patterns. Interested persons may contact the corresponding author for these simulated results.

## Conclusions

This research proposes a new nonparametric test for the ordered alternative problem. The new test statistic is based on the calculating all $k\left(x_{1j_{1}},x_{2j_{2}},\ldots ,x_{kj_{k}}\right)$ in proper (ascending) order. In other words, the new test statistic collects the information of each observation for each treatment to provide the message of “increasing” to the test statistics. A higher test statistics means a stronger “increasing” message. This is also why we expect the new test statistics to offer better power under certain situations.

Due to the small number of groups and sample sizes, we tabulated and listed their distribution as well as the exact mean and variance of the null distribution. From the equation for the exact mean and variance of the null distribution was derived and the asymptotic null distribution is normal were given.

We also use the example of ordinal risk prediction of colon cancer to compare the test statistics mentioned in the papers. A finite sample simulation study was also used to explore in-depth how the powers of JT, MJT, TM, KTP and KTMB tests under different underlying populations, treatment numbers and sample sizes. Based on the example and simulation results, we conclude that these tests frequently detect an ordered trend when, in fact, one does not exist. However, the KTMB test can reduce the error rate, at least not to the extent in which the JT and MJT tests do.

Ben Van Calster et. al. extend the main measure of binary discrimination, the c-statistic or area under the ROC curve, to nominal polytomous settings by polytomous discrimination index (PDI) [24]. They mention it is desirable that the risk of each group is highest for the case that belongs to this group in a set of cases. Therefore, the PDI score awarded to a set equals the number of groups for which this holds. Based on this point of view, in our opinion, the KTMB test can not only be used for detecting the non-decreasing alternatives but can also be measured to summarize polytomous discrimination.

## Competing interests

The authors declare that they have no competing interest.

## Authors’ contributions

CHC designed the study, prepared the manuscript and performed statistical analyses. WY drafted and assisted with the revision of the article. CCC and YYH participated in and carried out the field work. All authors read and approved the final manuscript.

## Pre-publication history

The pre-publication history for this paper can be accessed here:

http://www.biomedcentral.com/1471-2288/13/148/prepub

## Supplementary Material

Additional file 1 **Algorithm for computing **$\sum_{i=1}^{k-1}v_{i}^{2}$**.** Click here for file
