# Supplementary material for: Using the Bernoulli trial approaches for detecting ordered alternatives
Source: BMC Med Res Methodol. 2013 Dec 5;13:148. doi: 10.1186/1471-2288-13-148 (PMC3878968; doi:10.1186/1471-2288-13-148)
Supplement: Additional file 1 — Algorithm for computing ∑i=1k-1vi2. [file 1471-2288-13-148-S1.doc]

**Additional file 1 - Algorithm for computing** $\sum_{i=1}^{k-1} v_{i}^{2}$

Consider the case of i ties. Let l_0_ = 0 and $C_{0}^{0}=1$. Then under H_0_,

$$v_{i}^{2}=$$

$$c_{uv}=$$

$$[k-t_{1}-t_{2}-t_{3}-t_{4}-I(m\neq l_{1})\cdots I(m\neq l_{i})]!$$

$$[k-t_{1}-t_{2}-t_{3}-t_{4}-$$

$$I(n\neq l_{1})\cdots I(n\neq l_{i})]!$$

$$(t_{1}+t_{2}+t_{3}+t_{4})!$$

$$C_{t_{1}+t_{2}+t_{3}+t_{4}}^{I_{SS}(u,v)+I_{SL}(u,v)+I_{LS}(u,v)+I_{LL}(u,v)}$$

$$C_{w}^{I_{SS}(u,v)-t_{1}}C_{m-1-I(l_{1}<m)-\cdots-I(l_{i}<m)-w}^{I_{SL}(u,v)-t_{2}}$$

$$C_{x}^{I_{SS}(u,v)-t_{1}-w}C_{n-1-I(l_{1}<n)-\cdots-I(l_{i}<n)-x}^{I_{LS}(u,v)-t_{3}}$$

$$C_{y}^{I_{LS}(u,v)-t_{3}-[n-1-I(l_{1}<n)-\cdots-I(l_{i}<n)-x]}C_{k-m-I(l_{1}>m)-\cdots-I(l_{i}>m)-y}^{I_{LL}(u,v)-t_{4}}$$

$$C_{z}^{I_{SL}(u,v)-t_{2}-[m-1-I(l_{1}<m)-\cdots-I(l_{i}<m)-w]}$$

$$C_{k-n-I(l_{1}>n)-\cdots-I(l_{i}>n)-z}^{I_{LL}(u,v)-t_{4}-[k-m-I(l_{1}>m)-\cdots-I(l_{i}>m)-y]}$$

$$-\frac{1}{k^{2}}$$

Hence, we define

*I_SS_*(*u, v*), *I_SL_*(*u, v*), *I_LS_*(*u, v*), and *I_LL_*(*u, v*) = The numbers of *{1 : u-1}∩{1 : v-1}*, *{1 : u-1}∩{v+1 : 2k-i}*, *{u+1 : 2k-i}∩{1 : v-1}*, and *{u+1 : 2k-i}∩{v+1 : 2k-i}*.

*t_1_*, *t_2_*, *t_3_*, and *t_4_* = the numbers of ties from *I_SS_*(*u, v*), *I_SL_*(*u, v*), *I_LS_*(*u, v*), and *I_LL_*(*u, v*).

If *b* > *a* in $C_{b}^{a}$, *v_uv_* = 0.

In displays (1) and (2), we present formulas for the mean and variance of test statistic under H_0_. The proof of V_0_(T) is not presented for the sake of brevity as the arguments are similar to those presented for the case of ‘‘exactly three ties’’ in the proof of Theorem 1. Moreover, we illustrate a direct calculation of E_0_(T) and V_0_(T) in the particular case of $k=$3, and $n_{i}=$1, 2, or 3 in Table 1. Note that $(n_{1},$ $n_{2},$ $n_{3})$= (2, 1, 1), E_0_(T) = 2 and V_0_(T) = 2.667, which is in agreement with what we obtain using equation (1) and (2), namely,

E_0_(T) = 2, and

$v_{0}^{2}=\left(1-\frac{1}{3}\right)\prod_{i=1}^{3} n_{i}=1.333$,

$\sum_{i=1}^{3-1} v_{i}^{2}=2\times1\times[\frac{1}{24}\times0!\times0!\times2!\times(C_{2}^{2}+C_{2}^{2})-\frac{1}{3^{2}}+\frac{1}{24}\times0!\times1!\times1!\times(C_{1}^{2}C_{1}^{1}+C_{1}^{1}C_{1}^{1})-\frac{1}{3^{2}}+\frac{1}{24}\times0!\times$ $1!\times1!\times(C_{1}^{2}C_{1}^{1}+C_{1}^{1}C_{1}^{1})-\frac{1}{3^{2}}+\frac{1}{24}\times1!\times0!\times1!\times(C_{1}^{2}C_{1}^{1}+C_{1}^{1}C_{1}^{1})-\frac{1}{3^{2}}+\frac{1}{24}\times1!\times1!\times1!\times(C_{1}^{1}C_{1}^{2}C_{1}^{1}+C_{1}^{1}C_{1}^{2}C_{1}^{1})$ $-\frac{1}{3^{2}}+\frac{1}{24}\times1!\times1!\times0!\times(C_{1}^{1}C_{1}^{1}+C_{1}^{2}C_{1}^{1})-\frac{1}{3^{2}}+\frac{1}{24}\times1!\times0!\times1!\times(C_{1}^{2}C_{1}^{1}+C_{1}^{1}C_{1}^{1})-\frac{1}{3^{2}}+\frac{1}{24}\times1!\times1!\times0!\times(C_{1}^{1}$ $C_{1}^{1}+C_{1}^{2}C_{1}^{1})-\frac{1}{3^{2}}+\frac{1}{24}\times1!\times1!\times1!\times(C_{1}^{3}C_{1}^{2}C_{1}^{1})-\frac{1}{3^{2}}]$,

$\sum_{i=1}^{3-1} v_{i}^{2}=2\times1\times[\frac{2}{24}\times2-\frac{1}{3^{2}}+\frac{1}{24}\times3-\frac{1}{3^{2}}+\frac{1}{24}\times3-\frac{1}{3^{2}}+\frac{1}{24}\times3-\frac{1}{3^{2}}+\frac{1}{24}\times4-\frac{1}{3^{2}}+\frac{1}{24}\times3-\frac{1}{3^{2}}+$ $\frac{1}{24}\times3-\frac{1}{3^{2}}+\frac{1}{24}\times3-\frac{1}{3^{2}}+\frac{1}{24}\times6-\frac{1}{3^{2}}]=0.667$,

$v_{k}^{2}=2\times6\times(\frac{1}{6}-\frac{1}{9})=0.667$,

V_0_(T) = 1.333+0.667+0.667=2.667.
